# Functional genomics meets human genetics

**DOI:** 10.1515/medgen-2022-2160

**Published:** 2022-11-29

**Authors:** Kerstin U. Ludwig, Malte Spielmann

**Affiliations:** Institute of Human Genetics, University Hospital Bonn, University of Bonn, Bonn, Germany; Institute of Human Genetics, University Hospital Schleswig-Holstein, University of Lübeck and Kiel University, Lübeck & Kiel, Germany

Functional genomics is a key emerging topic in human genetics. The aim of functional genomics is to identify the structure, properties, and function of genes and their regulatory elements, or that of any other genomic sequence with an intracellular functional role. The ultimate goal is to elucidate the function of all components of the (human) genome. Initially, functional genomics centered around efforts to (i) map genetic elements of different types, and (ii) functional “gene-by-gene” investigations. However, due to recent technological advances, in particular the introduction of array-based technologies and next-generation-sequencing (NGS), functional genomics has been extended towards the generation and integration of systematic data from genomic, epigenomic, and transcriptomic projects, with complementary expertise from the fields of computational data science and synthetic biology.

In parallel, the field of human genetics is also undergoing a fundamental transformation, since the identification of individual genetic variants has now become relatively routine, and can be performed on an unprecedented scale, with great speed and accuracy. Due to the ever decreasing cost of sequencing, within the context of human genetic research, the assessment of variants via whole genome sequencing (WGS) is increasing, and this is likely to transform the diagnostic process for genetic diseases in the foreseeable future. The challenge for the field is thus now shifting instead towards variant interpretation, a process which requires an understanding of the consequences of a given variant at the molecular, cellular, and organismal level. This is a challenging undertaking, since most candidate variants are rare and/or are located in regions whose molecular functions are poorly understood. To keep pace with the ever increasing number of identified variants, novel systematic high throughput functional approaches to interpretation are required. As outlined below, this issue describes the introduction of functional genomics into the field of human genetics in four key areas:

The majority of individual variants are located in non-coding regions of the human genome, which is unsurprising given that protein-coding gene sequences account for only a very small proportion of the total genomic space. In their article, *Kircher & Ludwig* provide insights into current efforts to annotate genetic variation in these non-coding regions, as well as strategies for functional follow-up. Increasingly, at-scale experimental and bioinformatic approaches are now being developed, not least as a result of the pressure to both understand the mechanisms of the large number of loci identified in genome-wide association studies of multifactorial diseases and to increase the diagnostic yield among patients with monogenic disease.

However, even in the case of a variant that resides in the coding region of a gene – a scenario in which interpretation is typically more straightforward – many variants are nonetheless classified as variants of uncertain significance (VUS), which limits both the diagnostic yield and the benefit to the patient. Typically, these variants have not been previously reported, are not listed in databases, and/or have conflicting data concerning their causative role. In their article, *Dace and Findlay* explain how saturation genome editing is a powerful tool for the generation of systematic variant-effect maps for all potential amino acid changes in established monogenic genes.

One major challenge in variant interpretation is the resolution at which phenotypic consequences can be monitored. The depth and type of the molecular read-outs are often insufficient in terms of capturing the functional effect of an individual variant. For example, the interpretability of expression data from bulk RNA-Seq is limited when the effect of a variant is subtle, or limited to a certain tissue or cell type. The introduction of single-cell sequencing now enables the detection of molecular consequences at an appropriate cellular resolution. In their article, *Sreenivasan et al*. provide an overview on this technology and its potential role in human genetics.

Finally, *no man is an island*, and this is particularly true for multicellular organisms, in which different cell types interact with one another during development, and in both normal and disease conditions. To capture the full phenotypic consequences of genetic variants, systematic studies must be performed within the context of complex cellular interactions, with the most systematic approach being the investigation of entire organisms. Using genetically mediated kidney disorders as an example, the article by *Boettcher & Simons* describes the opportunities and challenges of different model organisms in terms of the functional follow-up of causal variants, and in understanding their effect at the organismal level.

We hope that these four selected topics provide insights into how functional genomic approaches may influence human genetics in the years to come. Notably, this issue of the *Medizinische Genetik* complements a series of previous issues on the challenges and limitations of NGS (02/2019), the annotation of non-coding variants for clinical use (02/2021), and the modeling of genetic disorders in cellular systems (03/2021). We refer interested readers to those issues of the *Medizinische Genetik* for complementary information.

